# Outbreak investigation in cargo ship in times of COVID-19 crisis, Port of Santos, Brazil

**DOI:** 10.11606/s1518-8787.2020054002461

**Published:** 2020-03-25

**Authors:** Eder Gatti Fernandes, Janice da Silva Santos, Helena Keico Sato

**Affiliations:** I Centro de Vigilância Epidemiológica “Prof. Alexandre Vranjac” Coordenadoria de Controle de Doenças Secretaria de Estado da Saúde de São Paulo Brasil Divisão de Imunização, Centro de Vigilância Epidemiológica “Prof. Alexandre Vranjac”, Coordenadoria de Controle de Doenças da Secretaria de Estado da Saúde de São Paulo Brasil; II Centro de Vigilância Epidemiológica “Prof. Alexandre Vranjac” Coordenadoria de Controle de Doenças Secretaria de Estado da Saúde de São Paulo Brasil Grupo de Vigilância Epidemiológica Santos - GVE XXV, Centro de Vigilância Epidemiológica “Prof. Alexandre Vranjac”, Coordenadoria de Controle de Doenças da Secretaria de Estado da Saúde de São Paulo Brasil; III Centro de Vigilância Epidemiológica “Prof. Alexandre Vranjac” Coordenadoria de Controle de Doenças Secretaria de Estado da Saúde de São Paulo Brasil Centro de Vigilância Epidemiológica “Prof. Alexandre Vranjac”, Coordenadoria de Controle de Doenças da Secretaria de Estado da Saúde de São Paulo Brasil

**Keywords:** Coronavirus Infections, epidemiology, Coronavirus Infections, prevention & control, Ships, International Health Regulations

## Abstract

In February 2020, a Chinese cargo ship docked at the Port of Santos with reports of crew members with a feverish and respiratory condition. A team was gathered to verify the existence of suspected cases of COVID-19 inside the vessel and define its clearance. All 25 crew members were interviewed, and no suspected cases were found. The vessel was then cleared for port activities. The investigation resulted from the implementation of the contingency plan to face a public health emergency of international importance and several surveillance entities cooperated.

## INTRODUCTION

On February 14, 2020, a shipping agency requested the Certificate of Free Pratique (operating license) to the Port of Santos, Brazil, for a cargo ship with a Hong Kong flag and 25 crew members on board. The expected arrival at the port was February 16, 2020. Among the presented documentation was the Declaration of Health and the Medical Logbook, in which two recent health occurrences were identified: 1) Chinese crew member with sore throat and cough; 2) Singaporean crew member with fever.

The ship’s last destination had been the Port of Singapore on January 21, 2020. In the last 30 days, the ship had also docked in the Port of Hong Kong (January 23) and three other Chinese ports: Yantian (January 22), Ningbo (January 19) and Shanghai (January 17). On January 28, 2020, China was considered an area of free transmission of the new coronavirus, which causes the COVID-19 disease. Anyone with respiratory symptoms and fever, and who had been in China in the 14 days prior to the onset of symptoms was considered a suspected case^1 , 2^ .

COVID-19 has been considered a public health emergency of international importance by the World Health Organization since the end of January 2020^1^ . Given the recent passage in the circulation area of new coronavirus and for having symptomatic crew members, the national and state Emergency Operations Committees (EOC) were activated. Then a field team with professionals from the Brazilian Health Regulatory Agency (Anvisa), the Health Surveillance Center (CVE, *Centro de Vigilância Epidemiológica* in Portuguese) of the state of São Paulo, the Health Surveillance Group (GVE, *Grupo de Vigilância Epidemiológica* in Portuguese) of the region of Santos and Health Surveillance of the municipality of Santos was mobilized. The aim of this study was to verify the existence of cases of coronavirus inside the vessel and define its operating license in the port of Santos, Brazil.

## METHODS

A descriptive study was conducted involving the 25 crew members of the vessel. The ship arrived at the Port of Santos on February 19, 2020, three days later than planned. The vessel was not authorized to operate, and no one was able to land. A team comprised of five technicians (two CVE physicians, a GVE nurse and two Anvisa ship inspection technicians) boarded with contact and respiratory personal protective equipment (PPE) for aerosols^1 , 2^ .

Initially, the two crew members reported as symptomatic were interviewed. Subsequently, the other crew members underwent the same procedure. The interview followed the list of crew members previously provided by the vessel’s command and was guided by a semi-structured questionnaire with identification variables, date and place of crew integration, internal function, accommodation, landing locations, previous symptoms, performed treatments and history of isolation. After each interview, the crew temperature was measured with a non-contact forehead thermometer.

While the investigation was taking place, a nurse from the health surveillance of the municipality of Santos waited outside the vessel equipped with PPE and material for collecting airway samples (swab and saline solution)^1 , 2^ . Also, an ambulance was prepared for displacing crew members to a hospital service if necessary.

A suspected case was defined as anyone who presented fever and at least one respiratory symptom (runny nose, sore throat, cough or dyspnea) from January 17, 2020 (date of passage by the Port of Shanghai) and up to 14 days after being in China. Given the identification of a crew member who fit the current definition of a suspicious case, airway samples would be collected from all crew members who presented feverish symptoms or any respiratory symptoms at the time of research. The collected samples would be sent to the Adolfo Lutz Institute, the public health laboratory of the state of São Paulo, for a viral panel (tests for identification of respiratory viruses), including the reverse transcription polymerase chain reaction (RT-PCR) for COVID-19. The identification of a suspicious case as of January 17, 2020 would result in the non-release of the operating license, that is, there would be no boarding or landing of crew or cargo.

## RESULTS

The first symptomatic crew member was male, 37 years old, born in Hunan, China, and joined the crew at the Port of Yantian, in China on January 21, 2020. He was the vessel’s chief officer and usually had contact with the entire crew. His first sign of the virus was a sore throat on January 29, 2020, which evolved to a cough and left conjunctival hyperemia the next day, with no fever. He received oral amoxicillin and ciprofloxacin with gentamicin in eye drops. The symptoms lasted 14 days and the subject was in isolation during the period. He presented no complaints during the investigation, with a temperature of 35.5°C.

The second symptomatic crew member was a 50-year-old male from Bangkalan, Indonesia, and joined the crew at the Port of Singapore on January 26, 2020. He was an oiler and stayed in the engine room for most of his working time. He had a fever on February 10, which lasted three days, and denied other symptoms. He received only antipyretic medication (paracetamol) and progressed to cure.

The crew consisted of 23 other members, all male, aged between 24 and 54 years. Most were from India (8), followed by Indonesia (5), Sri Lanka (4), Myanmar (3) and Bangladesh (1). Besides the two symptomatic crew members, four other boarded in 2020. Three crew members who were already on board landed at Chinese ports and had contact with the local population. The others remained on the vessel. All other 23 crew members interviewed denied any symptoms in the last two months. The temperatures measured ranged from 35.5 to 36.4°C. Because no crew member fit the definition of suspect case, no biological samples were collected.

## DISCUSSION

Since January 30, 2020, the World Health Organization has declared outbreak of acute respiratory disease with the new coronavirus, SARS-CoV-2, as a public health emergency of international importance^1 , 2^ . As such, all countries should be prepared to contain the spread of the virus by active surveillance with early detection, isolation and proper case management, investigation and timely notification. Therefore, the contingency plan for human infection with SARS-CoV-2 in the state of São Paulo stated that no vessel with any atypical report in its Declaration of Health or medical logbook would receive the Certificate of Free Pratique at the Port of Santos^2^ . This study showed an investigation resulting from this awareness of surveillance in the Port of Santos.

The air and sea transport system is involved in the spread of diseases worldwide, such as measles, influenza and coronavirus^3 , 4^ . Aircraft and vessels at maximum capacity, in confinement condition, facilitates the dissemination from person to person by droplets and aerosols, besides introducing new viruses in new areas after landing^3 , 4^ . But unlike cruise ships^4^ , there is no recent record of the involvement of cargo ships in the spread of the disease^3 , 5^ .

For being a new disease, susceptibility to COVID-19 is 100%. The entry of a single case in the vessel would lead to transmission to the entire crew. The investigation was justified for the following reasons: 1) passing of the vessel by Chinese ports; 2) two recorded health occurrences; 3) first symptomatic case from an area of COVID-19 transmission (China); 4) because medical assistance on a cargo ship is suboptimal^5^ ; 5) risk of introducing the disease in Brazil by the Port of Santos. The investigation also validated a method of objective approach in respiratory isolation situation for aerosols and contact.

The two symptomatic cases did not fit the definition of suspect of COVID-19. The other crew members did not show any symptoms or fever at the time of the investigation. For lack of evidence of an outbreak of respiratory transmission disease, the ship received its Certificate of Free Pratique at the port of Santos. The collaboration between the various surveillance entities (Anvisa, CVE, GVE and the municipality of Santos) was important to enable the investigation of the outbreak.
