# Supplementary material for: Outbreak investigation in cargo ship in times of COVID-19 crisis, Port of Santos, Brazil
Source: Rev Saude Publica. 2020 Mar 25;54:34. doi: 10.11606/s1518-8787.2020054002461 (PMC7100949; doi:10.11606/s1518-8787.2020054002461)
Supplement: Translation

# Translation: Investigação de surto em navio de carga em tempo de COVID-19, Porto de Santos, Brasil

**Authors:** Eder Gatti Fernandes, Janice da Silva Santos, Helena Keico Sato

**Affiliations:** I Brasil Divisão de Imunização, Centro de Vigilância Epidemiológica “Prof. Alexandre Vranjac”, Coordenadoria de Controle de Doenças da Secretaria de Estado da Saúde de São Paulo Brasil; II Brasil Grupo de Vigilância Epidemiológica Santos - GVE XXV, Centro de Vigilância Epidemiológica “Prof. Alexandre Vranjac”, Coordenadoria de Controle de Doenças da Secretaria de Estado da Saúde de São Paulo Brasil; III Brasil Centro de Vigilância Epidemiológica “Prof. Alexandre Vranjac”, Coordenadoria de Controle de Doenças da Secretaria de Estado da Saúde de São Paulo Brasil

**Keywords:** Infecções por Coronavirus, epidemiologia, Infecções por Coronavirus, prevenção & controle, Navios, Regulamento Sanitário Internacional

## Abstract

Em fevereiro de 2020, um navio de carga vindo da China atracou no Porto de Santos com relato de tripulantes com quadro febril e respiratório. Uma equipe foi mobilizada para verificar a existência de casos suspeitos de COVID-19 dentro da embarcação e definir a liberação da embarcação no porto. Todos os 25 tripulantes foram entrevistados e não foram encontrados casos suspeitos. Então a embarcação foi liberada para atividades no porto. A investigação resultou da aplicação do plano de contingência diante de uma emergência de saúde pública de importância internacional e houve a colaboração de diversas entidades de vigilância.

## INTRODUÇÃO

No dia 14 de fevereiro de 2020, uma agência marítima solicitou o Certificado de Livre Prática (autorização para operar) ao Porto de Santos, Brasil, para um navio cargueiro com bandeira de Hong Kong e 25 tripulantes a bordo. A previsão de chegada ao porto era 16 de fevereiro de 2020. Entre a documentação apresentada, havia a Declaração Marítima de Saúde e o Diário de Bordo de Saúde. Neles foram identificadas duas ocorrências em saúde recentes: 1) tripulante natural da China com dor de garganta e tosse; 2) tripulante natural de Singapura com febre.

O último destino do navio havia sido o porto de Singapura, em 21 de janeiro de 2020. Nos últimos 30 dias, o navio havia atracado também no porto de Hong Kong (23 de janeiro) e em outros três portos da China: Yantian (22 de janeiro), Ningbo (19 de janeiro) e Xangai (17 de janeiro). Em 28 de janeiro de 2020, a China foi considerada área de livre transmissão do novo coronavírus, que provoca a doença COVID-19 (do inglês *coronavirus disease 2019* ). Qualquer pessoa com sintomas respiratórios e febre e que tenha estado na China nos 14 dias prévios ao início dos sintomas era considerada caso suspeito^1 , 2^ .

A COVID-19 é considerada emergência de saúde pública de importância internacional pela Organização Mundial de Saúde desde o final do mês de janeiro de 2020^1^ . Diante da recente passagem em área de circulação do novo coronavírus e por ter sintomáticos dentro do navio, os Comitês de Operações de Emergência (COE) nacional e estadual foram acionados. Então uma equipe de campo com profissionais da Agência Nacional de Vigilância Sanitária (Anvisa), Centro de Vigilância Epidemiológica (CVE) do estado de São Paulo, Grupo de Vigilância Epidemiológica de Santos (GVE) e Vigilância Epidemiológica do Município de Santos foi mobilizada. O objetivo do estudo foi verificar a existência de casos de infecção pelo novo coronavírus dentro da embarcação e definir a autorização de livre prática da embarcação no porto de Santos, Brasil.

## MÉTODOS

Foi realizado um estudo descritivo envolvendo os 25 tripulantes da embarcação. O navio chegou ao porto de Santos no dia 19 de fevereiro de 2020, três dias após o previsto. A embarcação não recebeu autorização para operar e ninguém pôde desembarcar. Uma equipe composta por cinco técnicos (dois médicos do CVE, uma enfermeira do GVE e dois técnicos de inspeção de navio da Anvisa) subiram a bordo com equipamentos de proteção individual (EPI) de contato e respiratório para aerossóis^1 , 2^ .

Inicialmente foram entrevistados os dois tripulantes reportados como sintomáticos. Posteriormente foram entrevistados os demais tripulantes. A entrevista seguiu a lista de tripulantes fornecida previamente pelo comando da embarcação e foi guiada por questionário semiestruturado com variáveis de identificação, data e local de incorporação à tripulação, função interna, alojamento, locais de desembarque, sintomas prévios, tratamentos realizados e histórico de isolamento. Após cada entrevista, a temperatura do tripulante foi aferida com termômetro de testa sem contato.

Enquanto acontecia a investigação dentro da embarcação, uma profissional de enfermagem da vigilância do município de Santos aguardava fora da embarcação devidamente paramentada com EPI e com material para coleta de amostras de vias aéreas ( *swab* e solução salina)^1 , 2^ . Havia também uma ambulância preparada para deslocamento de tripulantes para um serviço hospitalar, caso fosse necessário.

Definiu-se como caso suspeito o indivíduo que apresentou febre e pelo menos um sintoma respiratório (coriza, dor de garganta, tosse ou dispneia) a partir do dia 17 de janeiro de 2020 (data da passagem pelo porto de Xangai) e até 14 dias após ter estado na China. Diante da identificação de tripulante que se encaixasse na definição corrente de caso suspeito, amostras de vias aéreas superiores seriam coletadas de todos os tripulantes que apresentassem quadro febril ou qualquer sintoma respiratório no momento da investigação. As amostras coletadas seriam encaminhadas ao Instituto Adolfo Lutz, o laboratório de saúde pública do estado de São Paulo, para realização de painel viral (exames para identificação de vírus respiratórios), incluindo a *reverse transcription polymerase chain reaction* (RT-PCR) para COVID-19. A identificação de caso suspeito a partir do dia 17 de janeiro de 2020 resultaria na não liberação da autorização de livre prática, ou seja, não haveria embarque ou desembarque de tripulantes ou carga.

## RESULTADOS

O primeiro tripulante sintomático era do sexo masculino, 37 anos de idade, natural de Hunan, China, e incorporou-se à tripulação no porto de Yantian, também na China em 21 de janeiro de 2020. Sua função era de diretor ( *chief officer* ) da embarcação e normalmente tinha contato com toda a tripulação. Ele iniciou o quadro com dor de garganta em 29 de janeiro de 2020 e evoluiu com tosse e hiperemia conjuntival à esquerda no dia seguinte. Não apresentou febre. Recebeu amoxicilina via oral, além de ciprofloxacina com gentamicina em colírio. Os sintomas duraram 14 dias e o indivíduo ficou em isolamento durante o período. Apresentou-se sem queixas durante a investigação, com temperatura aferida de 35,5 °C.

O segundo tripulante sintomático também era do sexo masculino, 50 anos de idade, natural de Bangkalan, Indonésia, e incorporou-se à tripulação no porto de Singapura em 26 de janeiro de 2020. Sua função era de engraxador e ficava na sala de máquinas na maior parte do seu tempo de trabalho. Ele apresentou febre em 10 de fevereiro, que durou três dias. Negou outros sintomas. Recebeu apenas medicação antitérmica (paracetamol) e evoluiu para a cura.

A tripulação era composta por outros 23 tripulantes, todos do sexo masculino, com idade entre 24 e 54 anos. A maioria dos tripulantes era natural da Índia (8), seguida de Indonésia (5), Sri Lanka (4), Myanmar (3) e Bangladesh (1). Além dos dois tripulantes sintomáticos, outros quatro tripulantes embarcaram em 2020. Três tripulantes que já estavam a bordo desembarcaram em portos da China e tiveram contato com pessoas locais. Os demais permaneceram na embarcação. Todos os outros 23 tripulantes entrevistados negaram qualquer sintoma nos últimos dois meses. As temperaturas aferidas variaram de 35,5 a 36,4 °C. Por não haver tripulante que se encaixasse na definição de suspeito, nenhuma amostra biológica foi coletada.

## DISCUSSÃO

Desde 30 de janeiro de 2020, a Organização Mundial de Saúde declarou o surto de doença respiratória aguda com o novo coronavírus, o SARS-CoV-2, como uma emergência de saúde pública de importância internacional^1 , 2^ . Com isso, todos os países do mundo deveriam estar preparados para conter a disseminação do vírus por meio de vigilância ativa com detecção precoce, isolamento e manejo adequado dos casos, investigação e notificação oportuna. Por isso, o plano de contingência para infecção humana com o SARS-CoV-2 no estado de São Paulo definiu que não seria fornecido o Certificado de Livre Prática no Porto de Santos a uma embarcação com qualquer relato atípico na sua Declaração Marítima de Saúde ou no *medical logbook* ^2^ . O presente estudo mostrou uma investigação decorrente dessa sensibilização da vigilância no porto de Santos.

O sistema de transporte aéreo e marítimo está envolvido na disseminação de doenças pelo mundo, como sarampo, influenza e coronavírus^3 , 4^ . A lotação das aeronaves e embarcações, em condição de confinamento, facilita a disseminação de pessoa a pessoa por gotículas e aerossóis, além de introdução de novos vírus em novas áreas após o desembarque^3 , 4^ . Mas ao contrário dos navios de cruzeiro^4^ , não há registro recente do envolvimento de navios de carga na disseminação de doença^3 , 5^ .

Por ser uma doença nova, a suscetibilidade à COVID-19 é de 100%. A entrada de um único caso na embarcação levaria à transmissão a toda a tripulação. A investigação se justificou pelos seguintes motivos: 1) passagem da embarcação por portos chineses; 2) duas ocorrências de saúde registradas; 3) primeiro sintomático proveniente de área com transmissão de COVID-19 (China); 4) o fato de a assistência médica em um navio de carga ser subótima^5^ ; 5) risco da introdução da doença no Brasil pelo porto de Santos. Além disso, a investigação validou um método de abordagem objetiva em situação de isolamento respiratório para aerossóis e contato.

Os dois casos sintomáticos não se enquadraram na definição de suspeito de COVID-19. Os demais tripulantes não apresentaram qualquer sintoma nem febre no momento da investigação. Por não haver evidência de surto de doença de transmissão respiratória, o navio recebeu seu Certificado de Livre Prática no porto de Santos. A colaboração entre as diversas entidades de vigilância (Anvisa, CVE, GVE e município de Santos) foi importante para viabilizar a investigação do surto.
